# My path to citrin deficiency

**DOI:** 10.1002/jimd.12818

**Published:** 2024-11-24

**Authors:** John E. Walker

**Affiliations:** ^1^ Medical Research Council Mitochondrial Biology Unit University of Cambridge Cambridge UK

**Keywords:** citrin deficiency, cure, diagnosis, mitochondria, treatment, urea cycle

## Abstract

Citrin belongs to the SLC25 transport protein family found mostly in inner mitochondrial membranes. The family prototype, the ADP–ATP carrier, delivers ATP made inside mitochondria to the cellular cytoplasm and returns ADP to the mitochondrion for resynthesis of ATP. In pre‐genomic 1981, I noticed that the protein sequence of the bovine ADP–ATP carrier consists of three related sequences, each containing two transmembrane α‐helices traveling in opposite senses. Colleagues and I demonstrated that two other mitochondrial carriers had similar features. From emergent genomic sequences, it became apparent that they represented a large family of transport proteins with the same characteristic threefold repeats. The human genome encodes 53 members, but the functions of many were unknown. So, colleagues and I determined how to make these proteins in *Escherichia coli* and introduce them into liposomes to allow exploration of their transport functions. The 27 human family members to have been thus identified include citrin and the closely related protein aralar. Both exchange aspartate from the mitochondrial matrix for cytosolic glutamate plus a proton. Citrin is expressed predominantly in liver and non‐excitable tissues, whereas aralar is the dominant form in the brain. Each has a membrane extrinsic N‐terminal Ca^2+^‐binding domain, a transport domain, and a C‐terminal amphipathic α‐helix. Human mutations in citrin impair the urea cycle, malate–aspartate shuttle, gluconeogenesis, amino acid breakdown, and energy metabolism leading to citrin deficiency. Currently, the complex etiology of this condition is poorly understood and new knowledge would help to improve diagnosis, therapies, and finding a cure. My aims are to seek a basic understanding of the etiology of citrin deficiency and to use that knowledge in improving diagnostic procedures and in developing new treatments and a cure.

## 1 INTRODUCTION

In 1974, my life changed for the better when Frederick Sanger invited me to move from the Pasteur Institute in Paris, where I worked, to join his Division of Protein and Nucleic Chemistry at the Medical Research Council's Laboratory of Molecular Biology (LMB) in Cambridge. There, I helped Sanger by uncovering features in his revolutionary bacteriophage and mammalian mitochondrial DNA sequences by sequencing the encoded proteins directly.^1^, ^2^, ^3^ During the analysis of the mitochondrial genomes, I explored the literature on mitochondria extensively and became fascinated by the complicated multi‐protein enzymes that form the respiratory chain in the inner mitochondrial membrane. Therefore, in 1977, when Sanger suggested that I should develop my own independent research program, I decided to study the mitochondrial ATP synthase, with the long‐term aim of arriving at a deep understanding of how ATP was made from an intermediate energy form derived from the metabolism of carbohydrates, fats, and proteins in ingested foods, known as the transmembrane proton motive force.^4^ Sanger encouraged me to take on this daunting task which Max Perutz described to me as a life sentence, correctly as it turned out.

So, around 1978, began my 40‐year‐long journey toward understanding how ATP is made.^5^, ^6^ The astounding fact is that each day, from a steady state level in the human body of ATP of about 50 grams, every human being makes an amount of ATP approximately equivalent to their body weight in order to sustain their lives. The ATP synthase makes these ATP molecules from ADP and phosphate inside the mitochondria. A transport protein in the inner membranes of mitochondria known as the ADP–ATP translocase (or the ADP–ATP carrier) provides essential support by exporting each newly synthesized ATP molecule in exchange for an external ADP molecule that has arisen from the hydrolysis of ATP in life‐sustaining energy requiring processes. Another transport protein returns the phosphate to the mitochondrial matrix.

## 2 MITOCHONDRIAL TRANSPORT PROTEINS

**FIGURE 1:**
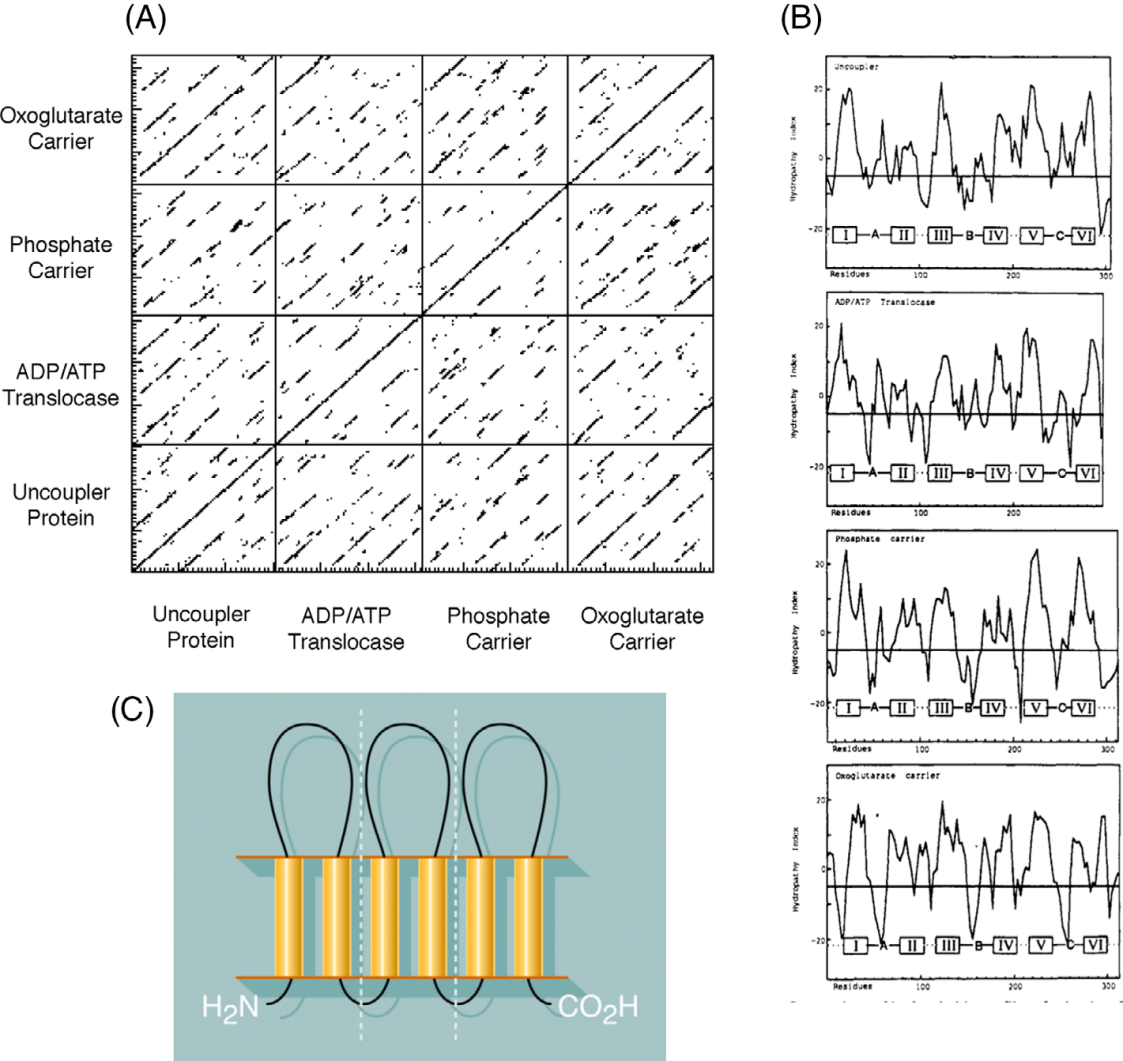
The discovery of the characteristic features of members of the family of transport proteins SLC25A. (A) The sequences of the ADP–ATP, phosphate, oxoglutarate–malate carriers, and the uncoupling protein UCP1 compared with themselves demonstrated the presence in each protein of three related sequences each about 100 amino acids in length. Pairwise comparisons demonstrated that all the repeats are related to each other.^9^ (B) The hydrophobic profiles of the four proteins suggested that all the repetitive elements consisted of two transmembrane α‐helices linked by a more hydrophilic sequence leading to a common model shown in (C). Today, we know that these features characterize all members of the SLC25A family.

One day in 1981, I noticed the publication of the protein sequence of the bovine ADP–ATP translocase determined by direct chemical sequencing of the protein,^7^ but the paper contained no comments on any possible significant features in the sequence. So, together with a visiting Finnish postdoctoral scientist, Matti Saraste, I applied a powerful bioinformatic tool called DIAGON^8^ to analyze the sequence of the translocase. DIAGON had been developed by a colleague, Andrew McLachlan, for analyzing relationships based on sequence similarities, both between different proteins and within the same protein. The astonishing result was that the approximately 300 residue ADP–ATP translocase protein is made of three related elements each about 100 amino acids long (Figure 1A). Then we applied another bioinformatic tool called HYDROPLOT^10^ to discover that all three repetitive elements consisted of two hydrophobic stretches of amino acids, each of an appropriate length to be folded into a trans‐membrane α‐helix, separated by a fairly extensive sequence of more polar amino acids (Figure 1B). On this basis, we proposed that the structure of the ADP–ATP carrier consisted of six transmembrane α‐helices with each pair of α‐helices separated by a membrane extrinsic hydrophilic region (Figure 1C).^11^ I was keen to explore whether other mitochondrial transport proteins, with similar apparent molecular weights to the ADP–ATP carrier, might also have comparable sequence features. So, next we, including Michael Runswick and a visiting Swedish post‐doctoral scientist, Pål Nyren, determined the sequence of the mitochondrial phosphate transporter.^12^ We found that its sequence also consisted of three related elements of approximately 100 amino acid residues, with each repetitive element capable of forming two hydrophobic trans‐membrane α‐helices, and the α‐helices again separated by a relatively extensive sequence of more polar amino acids. Moreover, the repeated elements in the phosphate carrier were related to the three repeated elements in the ADP–ATP carrier (Figure 1A,B). The publication of these activities prompted Professor Ferdinando Palmieri from the University of Bari, in Italy, to offer me a purified sample of another mitochondrial transport protein, the malate–oxoglutarate carrier, which, when we sequenced it in Cambridge, proved to have features similar to those that are characteristic of the ADP–ATP and phosphate carriers.^9^ They were also observed independently in the uncoupling protein, UCP‐1, responsible for heat generation by the mitochondria in brown adipose tissue^13^ (Figure 1A,B). A new family of transport proteins, now known as SLC25,^14^ had been discovered.

In the meantime, our studies of ATP synthases from bacteria, mitochondria, and chloroplasts continued apace, but we needed a supply of the constituent subunits, including membrane subunits to facilitate the reconstitution of domains of the mitochondrial enzyme, such as the features now known as the central and peripheral stalks.^15^, ^16^, ^17^ However, the recombinant expression of the membrane proteins proved to be toxic to strains of *Escherichia coli* commonly employed for this purpose. Therefore, a visiting French post‐doctoral scientist, Bruno Miroux, selected strains of *E. coli* that permitted the abundant expression of these hitherto toxic proteins in the form of insoluble inclusion bodies.^18^ However, not all membrane proteins were toxic in the original *E. coli* expression strain, and earlier, with Giuseppe Fiermonte, a post‐doctoral scientist visiting from the University of Bari, we had made the oxoglutarate–malate carrier in the form of inclusion bodies, solubilized them in a detergent, reintroduced the protein into lipid vesicles, and in Professor Palmieri's laboratory in Bari, Dr. Fiermonte had demonstrated that the bacterially expressed protein was competent in the transport of malate and oxoglutarate.^19^ The combination of expression of membrane proteins in the new bacterial strain together with the reconstitution and transport assay procedures has proved to be a powerful tool in this and other fields. Sometimes, it is referred to as EPRA (expression, purification, reconstitution, and assay). To date, 27 human family members and 20 yeast family members have been identified by this and related procedures, mostly in the laboratory of Professor Palmieri.^20^

Around the same time, in 1996, the sequence of the genome of *Saccharomyces cerevisiae* became available, followed in 2001 by the human genome sequence. The yeast genome encodes 35 proteins with the characteristic features of members of the SLC25 transporter family, and the human genome for 53 members, but, at the time, the functions of many of them were unknown.

## 3 IDENTIFICATION OF THE BIOCHEMICAL FUNCTION OF CITRIN

In 1999, Professors Kobayashi, Saheki and colleagues in Japan reported that the gene mutated in the urea‐cycle disorder known as adult‐onset type II citrullinemia (CTLN2), encodes a member of the mitochondrial carrier family, but its biochemical function was unknown.^21^ The protein, named citrin, was especially closely related to another member of the family, also of unknown function, expressed in excitable tissues, and known as aralar, as described in 1998 by Professor Satrústegui and her colleague Dr. del Arco in Madrid.^22^ In 2001, in an international collaboration between Professors Saheki, Satrústegui, Palmieri, myself, and our colleagues, it was demonstrated by the use of the EPRA procedure that the biochemical role of both citrin and aralar is to exchange aspartate from the mitochondrial matrix for cytosolic glutamate plus a proton^23^ in agreement with earlier studies on intact mitochondria.^24^ For this reason, citrin and aralar are known also as AGC2 and AGC1, respectively, where AGC denotes aspartate–glutamate carrier.

## 4 STRUCTURE AND MECHANISM OF CITRIN

**FIGURE 2:**
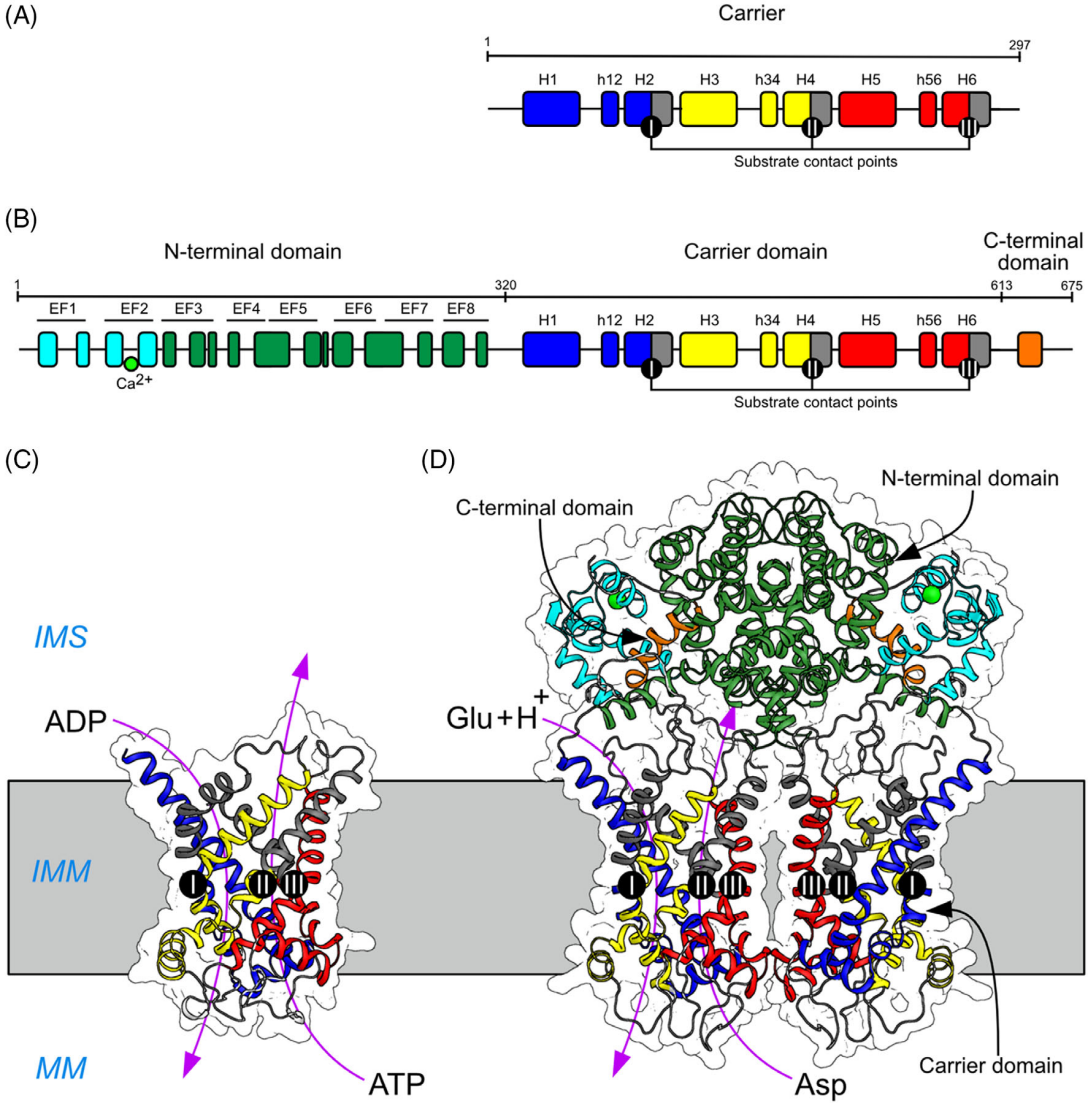
The structural domains of citrin and their functional elements. (A) The features of the carrier domain of citrin illustrating the α‐helical regions of each of the three repetitive elements described in Figure 1 in blue, yellow, and red. α‐Helices H1–H6 correspond to the six α‐helices in Figure 1. α‐Helices h12, h34, and h56 lie within the more hydrophilic regions connecting the pairs of α‐helices and were not predicted in Figure 1. They and the substrate contact points I–III were characterized by structural analysis.^26^ (B) The entire citrin molecule consists of three domains: The N‐terminal domain, the carrier domain, and the C‐terminal domain. The N‐terminal domain contains eight EF‐hands. EF‐hands 4–8 (green) comprise the static domain, shown in green, which is involved in the dimerization of citrin. EF3 provides a pivot point for movements that occur during transport. EF‐hands 1 and 2 (cyan) bind a calcium ion (green sphere). However, the transport activity is not regulated by the binding of Ca^2+^ as used to be thought.^27^ (C) Lateral view of the structure of prototypical SLC25A family member, the ADP–ATP carrier (PDB entries 4C9Q and 6GCI), viewed from inside the inner mitochondrial membrane (IMM), showing the repeated structural elements I‐III in blue, yellow, and red, respectively, and an ADP molecule in the intermembrane space (IMS) being exchanged to the mitochondrial matrix (MM) for an ATP molecule synthesized in the MM. (D) Model of the structure of citrin. Two N‐terminal domains interact in the IMS to dimerize the protein, whereas the two carrier domains are not in contact. They act independently of each other in the exchange of aspartate in the MM for glutamate plus a proton in the cytosol. Each C‐terminal domain consists of an amphipathic α‐helix which interacts with the N‐terminal domain in the same protomer (PDB entry 4P5W). PDB, protein database.

Citrin and aralar are atypical members of the SLC25 family^25^ as their structures, determined by Professor Edmund Kunji and colleagues,^26^ consist not just of the membrane intrinsic carrier domain, as in the prototypical ADP–ATP carrier, but they also have an extra N‐terminal domain, and a further C‐terminal domain consisting of an amphipathic α‐helix (Figure 2). Unlike all other family members which are monomeric,^28^ citrin and aralar are both homo‐dimeric proteins^26^ where the membrane extrinsic N‐terminal Ca^2+^‐binding domain with eight EF‐hands extends into the inter‐membrane space. The ATP magnesium‐phosphate transporter also has an additional membrane extrinsic N‐terminal Ca^2+^‐binding domain, but the protein is monomeric.^29^, ^30^ In citrin and aralar (but not in the monomeric ATP magnesium–phosphate transporter),^29^ the two N‐terminal domains interact with each other via EF‐hands 4–8 to form a static domain, with the two carrier domains not interacting and unassociated with each other. It used to be thought that this extrinsic domain was involved in the regulation of the transport activity of the protein by Ca^2+^, but recent work does not support this concept.^27^ Only EF‐hand 2 is occupied by a calcium ion, which is permanently bound under physiological conditions.^27^

The structures of the carrier domains of citrin and aralar are similar to that of the extensively studied monomeric ADP–ATP carrier.^28^, ^31^, ^32^, ^33^, ^34^, ^35^ They are 3‐fold pseudo‐symmetrical with three similar domains, corresponding to the three homologous sequence repeats (Figure 2).^26^ Each carrier domain consists of two transmembrane α‐helices linked by a loop containing a third α‐helix in the matrix, and the three domains are folded into a bundle of six α‐helices. The two individual carrier domains in the dimer cycle are independent of the other between two conformational states in a ping‐pong kinetic mechanism.^36^ According to this mechanism, the import of glutamate plus a proton and the export of aspartate occur consecutively by binding to a single central substrate‐binding site, which is alternately accessible from the two sides of the inner mitochondrial membrane.

## 5 CITRIN DEFICIENCY

**FIGURE 3:**
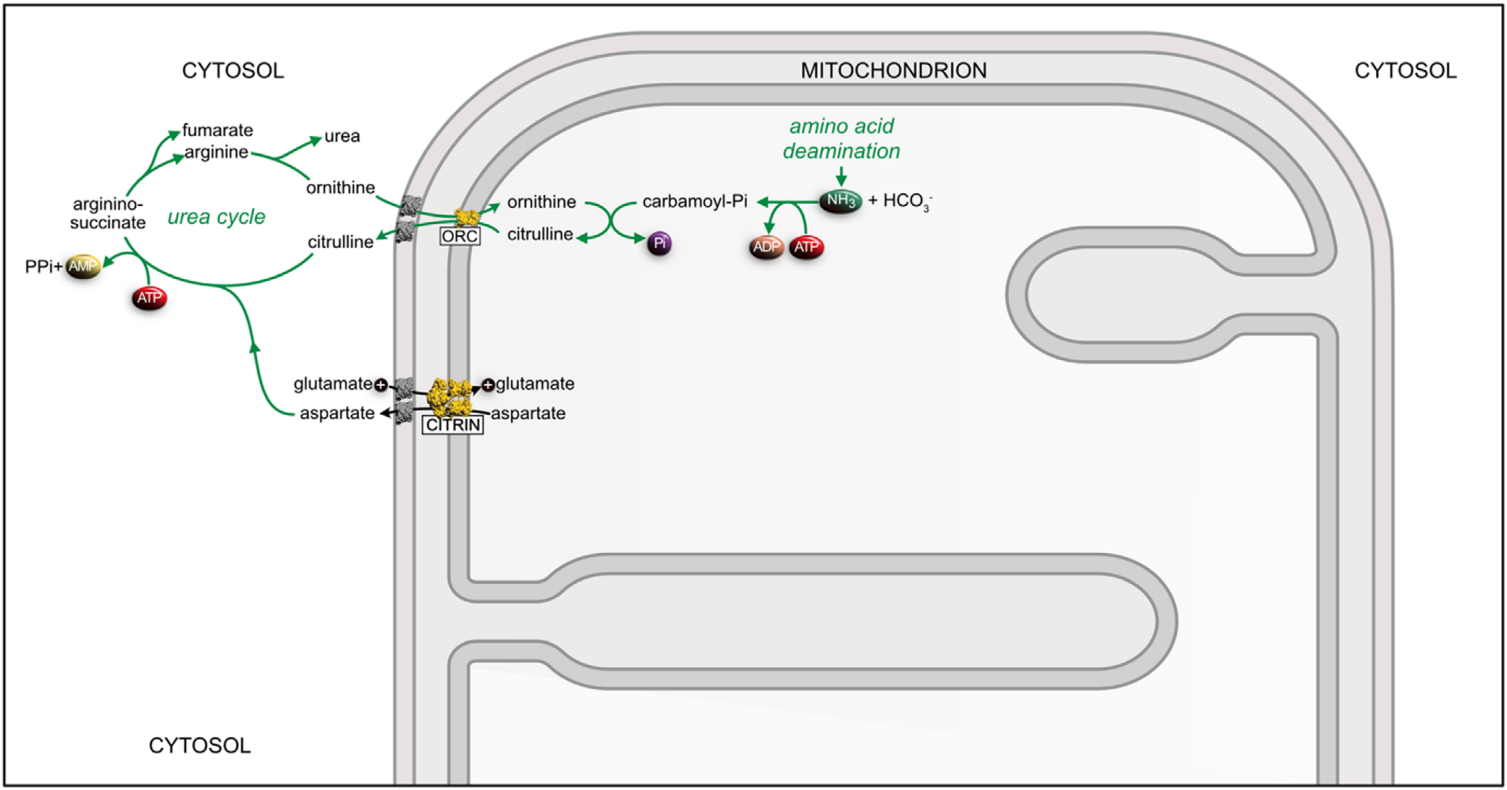
Participation of citrin in the urea cycle. Ammonia arising from the deamination of amino acids in the MM is combined with bicarbonate to form carbamoyl phosphate by carbamoyl phosphate synthase. Ornithine transcarbamoylase converts carbamoyl phosphate to citrulline, which is exchanged to the cytosol for external ornithine by the ornithine–citrulline carrier (ORC), another member of SLC25A. Citrin provides aspartate from the MM for the conversion of the external citrulline to argininosuccinate by argininosuccinate synthase. The remaining steps of the cycle consist of the conversion of argininosuccinate to arginine by the lyase, the release into the cytoplasm of urea generated in the conversion of arginine to ornithine by arginase, and the return of the resultant ornithine to the MM by the ORC. Substrates cross the outer mitochondrial membrane via the voltage‐gated anion channel shown in gray. When citrin is dysfunctional in CD, ammonia and citrulline accumulate leading to hyperammonemia and citrullinemia and the consequences associated with CD.

Citrin deficiency (CD) is a mitochondrial disease that gives rise to a defective urea cycle with three age‐related manifestations. Neonatal intrahepatic cholestasis caused by CD (NICCD), found in neonates and infants, is characterized by jaundice, failure to thrive, hypoproteinemia, hypoglycemia, multiple aminoacidemias including citrullinemia, and fatty liver.^37^, ^38^, ^39^ During childhood, the patients recover and go through an adaption stage with distinctive strong dietary preferences for high protein and high fat, and an aversion to carbohydrates, plus various other mild symptoms. At this stage, some suffer from a failure to thrive and dyslipidemia caused by CD, known as FTTDCD. In adulthood, between the ages of 20 and 50 years, a minority of patients develop citrullinemia type II (CTLN2), the most severe form of CD, which can lead to premature death. Recently, CTLN2 was renamed adult and adolescent citrin deficiency, or AACD.^40^ It is characterized by frequent bouts of hyperammonemia, liver steatosis, and hyperlipidemia leading to hepatic carcinoma, neuropsychiatric episodes, brain edema, and hyperlipidemia. An unexplained accompanying feature of CD is the liver‐specific decrease in argininosuccinate synthetase^41^ a key enzyme in the urea cycle (Figure 3). More than 60 years ago, when CD was described for the first time,^42^ it was thought to be confined mainly to East Asia and was studied especially in Japan where there is an estimated incidence of 1 in 17 000 based on carrier frequency of 1 in 65.^43^ Today, we know that CD is a pan‐ethnic disease that is increasingly being diagnosed in patients of non‐Asian origin.^43^

## 6 ROLES OF CITRIN AND METABOLIC CONSEQUENCES OF CD

The aspartate exported from mitochondria by citrin in the liver is required in the urea cycle for conversion in the mitochondrial inter‐membrane space of citrulline to argininosuccinic acid by argininosuccinate synthase (Figure 3). Thus, in patients with CD, the liver is incapable of forming urea to remove toxic ammonia generated by the metabolism of proteins in food. In consequence, many patients develop hyperammonemia and accumulate citrulline.

**FIGURE 4:**
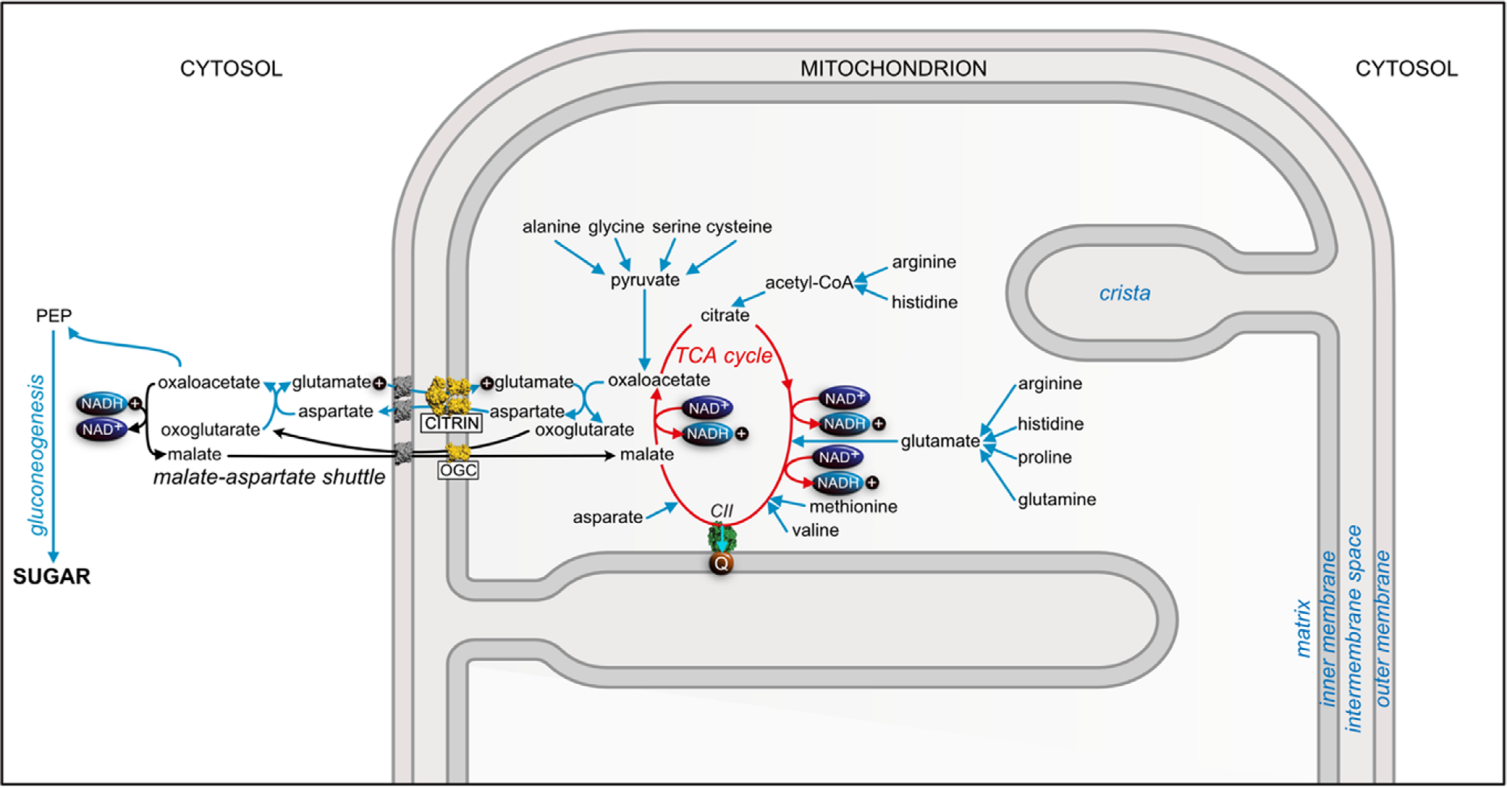
Participation of citrin in the aspartate–malate shuttle and gluconeogenesis. The aspartate–malate shuttle involves both citrin and the oxoglutarate–malate carrier (OGC). Reducing equivalents generated by glycolysis (see Figure 5) in the form of cytoplasmic NADH are transferred to the MM via intermediates in the tricarboxylic acid or TCA cycle. These reducing equivalents in the form of NADH in the MM enter the electron transport chain (not shown; see Figure 5) via complex I (NADH:Ubiquinone oxidoreductase). Other reducing equivalents generated in the TCA cycle enter the electron transport chain as reduced ubiquinone via complex II (CII; also known as succinate dehydrogenase). The metabolism of amino acids generates ammonia and provides intermediates that feed into the TCA cycle. When the cell's energy charge is low, and the concentration of ADP exceeds that of ATP, glucose is synthesized by gluconeogenesis from proteins via amino acids and pyruvate.

**FIGURE 5:**
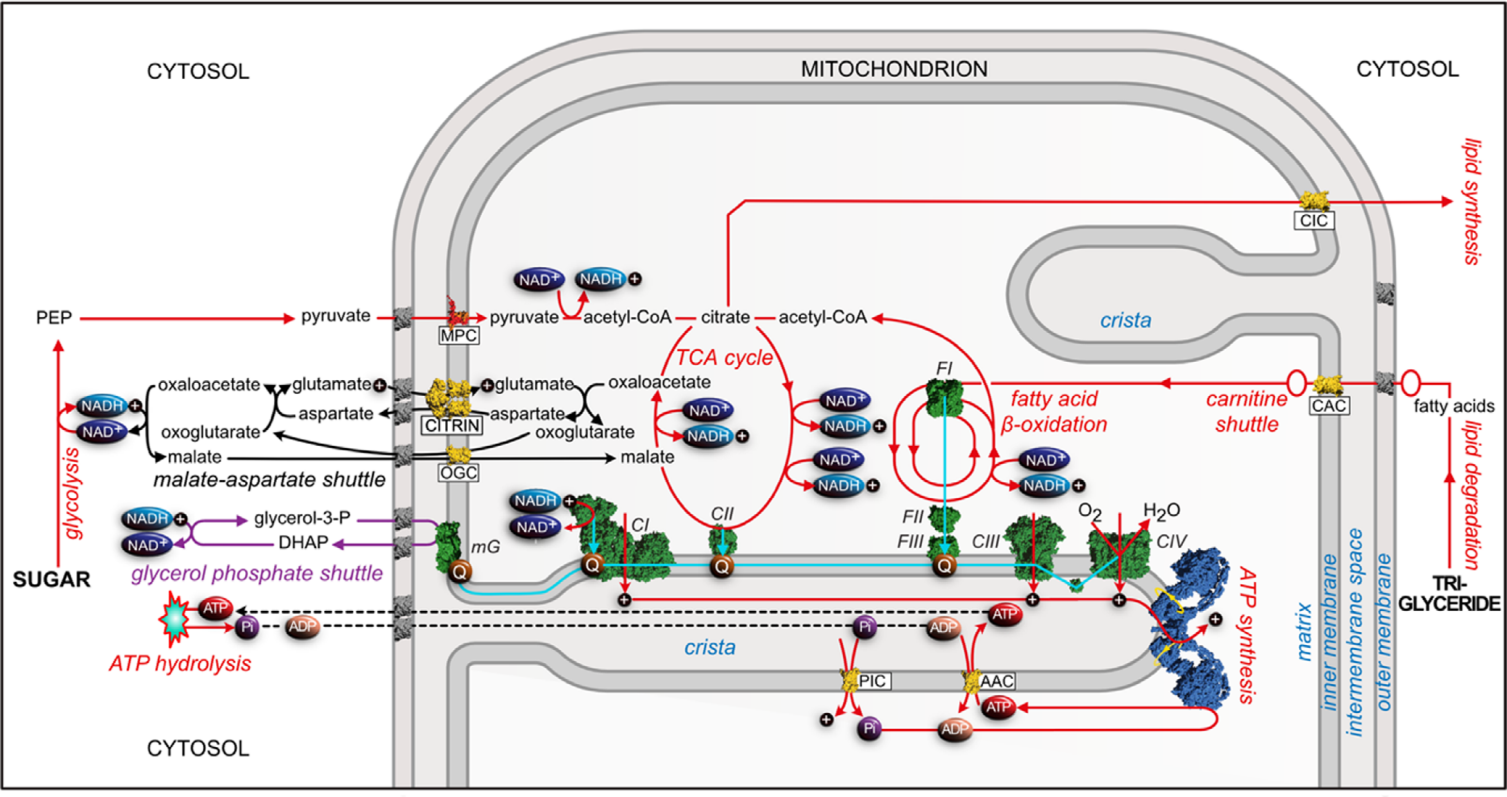
Roles of citrin in metabolism. In addition to its role in the urea cycle (not shown; see Figure 3), and via its involvement in the aspartate–malate shuttle (see Figure 4 also), citrin (shown in yellow, as are all members of SLC25 in the figure) influences the formation of ATP by the ATP synthase (blue) from ADP and phosphate (see legend to Figure 4). The ADP–ATP carrier (AAC) and the phosphate carrier (PIC) maintain the supply of ATP to the cytoplasm and ensure the resynthesis of the products of ATP hydrolysis. The synthesis of ATP is driven by proton motive force across the inner mitochondrial membrane generated by electron transfer via complexes I, III, and IV (CI, CIII, and CIV, respectively; green). The electron transfer chain (pale blue) is also sustained by electrons generated by the β‐oxidation of fatty acids involving acyl‐CoA dehydrogenases (FI). These electrons enter the reduced quinone pool in the IMM via the electron transfer flavoprotein (FII), and the ETF‐ubiquinone oxidoreductase (FIII). The glycerol phosphate shuttle (purple), operating via a cytosolic NAD^+^‐linked glycerol 3‐phosphate dehydrogenase (not shown) and a mitochondrial FAD‐linked glycerol 3‐phosphate dehydrogenase (mG), provides an alternative way of shunting reducing equivalents into mitochondria via reduced quinone in the IMM. Thus, in CD it could compensate for the dysfunctional malate–aspartate shuttle.

Another central biochemical function where citrin participates is the aspartate–malate shuttle, a biochemical device involving both citrin and the malate–oxoglutarate transporter for oxidizing cytosolic NADH produced by glycolysis and reducing NAD^+^ in the mitochondrial matrix (Figure 4). If citrin is absent or dysfunctional, glycolysis in the liver may be impaired, reducing both the flow of electrons into the electron transport chain via complex I, and the formation of ATP from carbohydrates. An alternative way of oxidizing NADH is provided by the glycerol phosphate shuttle (see Figure 5), but its lower activity in the human liver than in some other tissues (e.g., skeletal muscle and brain) may explain why patients with CD have a dietary preference for high fat and proteins over carbohydrates, as a functional malate–aspartate shuttle is not required for generation of energy from fat.

The liver is the main site of gluconeogenesis by which glucose is generated from non‐carbohydrate substrates such as pyruvate (under aerobic conditions), lactate (in the absence of oxygen), glucogenic amino acids, glycerol, and odd‐chain fatty acids arising from the breakdown of lipids. Gluconeogenesis in humans provides one of several ways of maintaining appropriate levels of glucose in the blood in order to avoid hypoglycemia, especially during overnight fasting when 90% of glucose is produced in the liver. As this pathway requires aspartate for conversion to oxaloacetate (see Figure 4), gluconeogenesis is impaired by dysfunctional citrin. Thus, during periods of fasting and exercise, patients may be unable to generate sufficient glucose, and hypoglycemia is a characteristic feature of many patients with NICCD during the adaptation stage.

Carbohydrate metabolism and lipid metabolism are connected as they both generate acetyl‐CoA for conversion to citrate. The mitochondrial citrate carrier (SLC25A1), also a member of the SLC25 family, exports citrate from the organelle thus providing a source of carbon and reducing equivalents for de novo lipogenesis. Therefore, impairment of sugar metabolism in CD patients could create an energy crisis by reducing de novo lipogenesis in adipose tissues and the replenishment of fat reserves. Dyslipidemia (high levels of lipids in the bloodstream) occurs in the adaption stage of CD, and fatty liver is often found in all stages, possibly because of the dietary requirements of CD patients, such as a high‐fat diet, although fatty liver can occur in infants with CD fed on breast milk or formula. Alternatively, it could be connected to the metabolic consequences of citrin dysfunction and other factors, such as liver failure, or both. It is notable that the peroxisome proliferator‐activated receptor‐α (PPAR‐α), the main regulator of fatty acid oxidation, is downregulated in patients.^44^ De novo lipogenesis is also important in the third trimester of pregnancy, when myelination of the developing central nervous system and deposition of body fat are required. Its impairment could lead to low birth weight and height.^45^, ^46^

## 7 GENETICS OF CD

The gene for human citrin is located at chromosome 7q21.3. It is inherited in an autosomal recessive manner and more than 100 different pathogenic mutations have been reported and reviewed.^43^ In the 53% of total cases of heterozygous CD patients, pathogenic alleles occur in several combinations, and about 42% of the remaining 47% of homozygous patients carry one specific mutation, namely c.851_854delGTAT. As discussed elsewhere,^27^ some mutations in the N‐terminal domain can disrupt at some point in the complex pathway of biogenesis and trafficking of citrin from its initial site of synthesis on cytoplasmic ribosomes to its fully folded and active state in the inner mitochondrial membrane, whereas mutations in the transport domain primarily affect the transport capability of the protein. The effects of these mutations on biogenesis especially require further study and elaboration. Such information could be helpful in arriving at an understanding of the etiology of CD which could then inform new potential routes to treatment. For example, it may be possible to correct defects in biogenesis, trafficking, and function of citrin by appropriate small molecule therapy, as employed, for example, to overcome some of the pathogenic mutations that lead to cystic fibrosis by affecting the localization and function of the cystic fibrosis transmembrane conductance regulator (CFTR).^47^

## 8 BETTER DIAGNOSIS AND THERAPIES, AND FINDING A CURE

CD can be difficult to recognize and diagnose in neonates, and therefore elevation of awareness of the condition, among medical practitioners especially, and improvement of its diagnosis are both desirable. The introduction of refined methods of newborn screening for inborn errors of metabolism has improved their detection^38^, ^48^ and it should improve the detection of CD also. Whole genome sequencing of neonates also offers substantial promise of improved diagnosis, and it is now being introduced, for example, by the National Health Service in the United Kingdom for the detection of more than 200 rare diseases, but not yet for CD.^49^ The consumption of dietary supplements such as medium chain triglycerides (MCTs) by patients appears to be beneficial.^50^ This favorable effect can be explained by the provision by MCTs of reducing equivalents via their cytosolic degradation to fatty acids, their uptake into mitochondria by the carnitine–acylcarnitine shuttle (CAC), and their entry into the β‐oxidation pathway. The consumption of sodium pyruvate as energy source for ameliorating CD has also been recommended.^51^ The consumption of nutraceuticals, such as nicotinamide riboside, for example, which is readily available and already approved for human consumption, has been reported to provide benefits in a cell model of CD.^52^ A 1:1 mixture of L‐ornithine and L‐aspartic acid (known as LOLA) has been advocated as a means of reducing circulating ammonia,^51^ as have ammonia scavengers such as sodium benzoate and phenylbutyrate.^51^, ^53^ However, there is still scope for developing better ammonia scavengers. Further assessment of the efficacy of all of these treatments for CD is needed in newer cell‐based models being developed at present, and in animal models of CD (see later). Objective‐controlled trials conducted in patient cohorts will also be required. Therefore, the building and the organization of such cohorts, and the characterization of each individual member are required in order to provide a sound basis for such studies.

A potential novel means of amelioration would involve the introduction into the liver of lipid nanoparticles^54^ containing molecules of the messenger RNA (mRNA) encoding wild‐type citrin. However, as the mRNA molecules and the encoded protein both have a limited duration, this treatment would have to be repeated over and over during the lifetime of the patient. Therefore, this approach provides amelioration and not a cure. It may eventually be possible to achieve a cure, however, by correcting the disease mutation by the rapidly evolving approaches of gene therapy.^55^

The development of these novel modes of amelioration and potential cure requires the establishment of benchmark criteria for success or failure of an intervention. It is also dependent upon the availability of appropriate model systems to test their efficacy before they can be approved for testing in humans. An early attempt has been made to develop a cell‐based model of CD,^52^ but better‐authenticated models are needed for the future. Therefore, new cellular models based on human hepatocytes are now being put in place and characterized, and the instigation of appropriate human liver spheroid and organoid models from induced pluripotent stem cells^56^ is also being considered. The value of this approach is illustrated by the successful reprogramming of somatic cells from a patient with a urea cycle defect, their genetic correction and differentiation into hepatic organoids, and the subsequent demonstration of genetic and phenotypic change in the edited cells consistent with the correction of the defect.^57^ Another tactic could be to try and follow an organ‐on‐a‐chip approach.^58^ Over the years, animal models have been developed successfully for many human diseases. Therefore, attempts have also been made to establish mouse models of CD by disruption of the murine citrin gene.^59^ These animals did not express citrin, but none of the phenotypic characteristics found in patients with NICCD or AACD were present, even when mice were aged up to 12 months. This lack of a CD phenotype was attributed to a high level of the glycerol phosphate shuttle (although it was present at the same high level in wild‐type animals). The glycerol phosphate shuttle can circumvent the disrupted malate–aspartate shuttle by providing an alternative means of shunting reducing equivalents into mitochondria (Figure 5). Therefore, a double knock‐out animal with the genes for both citrin and glycerol‐3‐phosphate dehydrogenase disrupted was generated and was found to recapitulate some features of CD.^60^ Some current activities are centered around the characterization of a citrin knock‐out rat model, and others the potential of replacing disfunctional citrin in the liver with aralar.^61^ As CD can be viewed as three different conditions, NICCD, the adaption stage, and AACD, in order to properly characterize and study the physiology and biochemistry of each stage it may be necessary to develop separate animal models for each of the three stages.

## 9 EXTENDING THE BASIC SCIENCE OF CD

As described earlier, our knowledge of the molecular structure and mechanism of citrin are well advanced.^26^, ^27^ An important genetic factor not so far considered in seeking an explanation of the complex etiology of CD is the possible involvement of genetic modifiers. Genetic modifiers are genetic variants that modify the phenotypic outcome of the primary disease‐causing variant. They act by either enhancing or suppressing the severity of the disease condition, but may not be disease‐causing themselves. Modifier variants can change the phenotype of a target gene by having a genetic, biochemical, or functional interaction with one or more target gene(s) or gene product(s). Well‐known examples are associated with Gaucher disease, cystic fibrosis, and von Hippel–Lindau syndrome^62^ and they help to explain the variable phenotypes of these conditions. Often, they are found in a functionally similar pathway to the target gene because of possible genetic interactions. Today, the most effective way of identifying genes associated with a particular disease is to use genome‐wide association studies (GWAS). By this approach, the entire genomes of a large group of people are searched for small variations in the form of single nucleotide polymorphisms (SNPs). In each study, hundreds or thousands of SNPs are examined at the same time to identify those found more frequently in people with a certain disease than in people without it. Such SNPs are said to be associated with the disease and they help to pinpoint genes that are likely to be involved in disease development. Once they become available, the whole genome sequences of patients can be analyzed for any such associations.

A related topic concerns the transcriptional regulation of expression of both citrin and aralar to try and arrive at an understanding of why citrin is expressed predominantly in non‐excitable tissues, such as liver, kidney, and pancreas, and aralar predominantly in the brain and other excitable tissues. The transcriptional promoter region of citrin has been investigated both in vitro and in vivo.^63^ It lacks a TATA box, which is a key element in the transcriptional initiation of many other eukaryotic genes, but it does contain a core promoter region CCAT box and a GC box, the presence of both of them being required for the expression of citrin. Two transcription factors, FOXA2 and USF1, have different roles in regulating the expression of citrin. FOXA2 is an activator in hepatic cells, and USF1 acts as a positive transcription factor that binds to the basal promoter ensuring expression of citrin in a range of tissues.^64^ Similar studies on aralar have not been reported. The aim of understanding this aspect of citrin and aralar is to provide a potential treatment for CD by stimulating the expression of aralar in the livers of patients to compensate for dysfunctional citrin. Such an approach is similar to that taken in the successful treatment of β‐thalassemias in adults by the administration of hydroxyurea, which stimulates in the adults the expression of the otherwise silent fetal globin gene.^65^ Other features that influence the expression of many other genes are G‐quadruplexes.^66^, ^67^ Currently, it is not known whether the genes for citrin and aralar harbor such features.

Another possible factor that could be part of the explanation of the complex etiology of CD is that the biochemical and physiological perturbations accompanying dysfunctional citrin may lead to the stimulation of compensatory biochemical pathways and physiological functions. Such mechanisms and other possibilities could become evident in metabolic, transcriptomic, and proteomic comparisons of wild‐type and citrin‐knockout cell lines, and materials from the livers of wild‐type and knock‐out animal models, for example, once they have been derived and authenticated.

Citrin is expressed not only in the liver but also in the pancreas, kidney, and heart. Thus, it is thought that the metabolic disorder arising with CD leads to continuously injured pancreatic acinar cells manifest in elevated levels in the serum of serine protease inhibitor Kazal type 1 (SPINK1), also known as the pancreatic secretory trypsin inhibitor (PSTI).^68^ Thus, in animal models of CD, it will be necessary to characterize the impact of disruption of the gene for citrin in not only the liver but also in these other tissues where citrin is expressed and CD has an additional impact.

## 10 PERSPECTIVES

In the 25 years since CD was first described, significant progress has been made toward a comprehension of its diversified phenotype based on biochemical and genetic studies. These advances have been and are being translated into improvements in the diagnosis of the condition and the treatment and care of patients. Future development of the investigation of this condition requires raising awareness of CD, promoting improvements in diagnosis and treatment, and developing a cure.

## AUTHOR CONTRIBUTIONS

The article was written by John E. Walker.

## FUNDING INFORMATION

My activities relating to citrin and citrin deficiency are funded by the Citrin Foundation Grant RG23006.

## CONFLICT OF INTEREST STATEMENT

The author declares no conflicts of interest.

## Data Availability

All data used in this review are accessible through the peer‐reviewed publications cited herein.
